# Molecular and cellular mechanisms underlying the evolution of form and function in the amniote jaw

**DOI:** 10.1186/s13227-019-0131-8

**Published:** 2019-08-12

**Authors:** Katherine C. Woronowicz, Richard A. Schneider

**Affiliations:** 1 Department of Orthopaedic Surgery, University of California at San Francisco, 513 Parnassus Avenue, S-1161, Box 0514, San Francisco, CA 94143-0514 USA; 2 Present Address: Department of Genetics, Harvard Medical School, Orthopaedic Research Laboratories, Children’s Hospital Boston, Boston, MA 02115 USA

**Keywords:** Amniote jaw development and evolution, Form and function, Neural crest, Secondary cartilage, Mechanical environment

## Abstract

The amniote jaw complex is a remarkable amalgamation of derivatives from distinct embryonic cell lineages. During development, the cells in these lineages experience concerted movements, migrations, and signaling interactions that take them from their initial origins to their final destinations and imbue their derivatives with aspects of form including their axial orientation, anatomical identity, size, and shape. Perturbations along the way can produce defects and disease, but also generate the variation necessary for jaw evolution and adaptation. We focus on molecular and cellular mechanisms that regulate form in the amniote jaw complex, and that enable structural and functional integration. Special emphasis is placed on the role of cranial neural crest mesenchyme (NCM) during the species-specific patterning of bone, cartilage, tendon, muscle, and other jaw tissues. We also address the effects of biomechanical forces during jaw development and discuss ways in which certain molecular and cellular responses add adaptive and evolutionary plasticity to jaw morphology. Overall, we highlight how variation in molecular and cellular programs can promote the phenomenal diversity and functional morphology achieved during amniote jaw evolution or lead to the range of jaw defects and disease that affect the human condition.

## Introduction

The jaws of amniotes display a marvelous array of sizes and shapes, and there are countless examples of how the form of the jaws has evolved to function in every conceivable ecological niche [1–7]. One obvious purpose for the jaw apparatus is to obtain, manipulate, process, and ingest dietary items. For instance, among reptiles, many snakes often consume prey larger than their own skulls and can accommodate extreme expansion with highly flexible upper and lower jaws. Large prey is incrementally forced down the esophagus by “snout shifting” or “pterygoid walking” in which tooth-bearing elements of the upper jaw alternately ratchet over the prey [8]. Additionally, while most amniote jaws are bilaterally symmetrical, snail-eating snakes (i.e., *Pareas*) have broken the symmetry of the dentition on their mandibles and develop more teeth on the right side as a means to prey upon clockwise-coiled (dextral) snails [9, 10]. Similarly, among birds, crossbills (i.e., *Loxia*) have bilaterally and dorsoventrally asymmetrical beaks such that the distal tips traverse one another. The lower jaw crosses to the left or right side with equal frequencies in crossbill populations [11] and this unusual adaptive co-evolution permits these birds to pry open conifer cone scales and extract seeds [12, 13]. Within mammals, giant anteaters (i.e., *Myrmecophaga*), which retrieve insects from tightly confined spaces like insect burrows, have evolved a specialized ability to “open” their jaws by rotating their mandibles along the long axis rather than by depressing the mandibles [14]. These are but a few extreme examples of what amniotes have accomplished with their jaws.

Yet while myriad jaw morphologies exist today and in the fossil record, all amniote jaws share common developmental and evolutionary origins, and their form and function are typically achieved by integrating many of the same adjoining skeletal, muscular, nervous, vascular, and connective tissue components [15, 16]. How then does the species-specific form of the jaws emerge in development and change during evolution in relation to function? In particular, what molecular and cellular mechanisms pattern the jaws of embryos in a manner that anticipates later adult use and promotes adaptation? These are fundamental questions in biology and there is a long history of efforts to answer them using the jaw complex as a subject of study.

Early attempts to link form and function in the jaws as well as the skull more broadly began at the gross anatomical level. Meticulous descriptions conducted in a transcendental and pre-evolutionary framework such as those from Goethe, Oken, Dumeril, Geoffroy, Owen, and many others laid the foundation for comparative methods to study morphological variation and adaptation [17–19]. Describing form and function among animals required special language, and Owen coined, “homology” and “analogy” with this goal in mind. Such concepts facilitated discussions about the structural plan for vertebrates and whether cranial elements being compared across taxa were indeed “the same organ in different animals under every variety of form and function” [20, p. 379]. In line with the transcendentalists before him, Owen postulated that the vertebrate skull and its constituent parts like the jaws extended as a serial homolog of the trunk skeleton [21, 22]. Owen’s ideas impacted the way the concept of homology and the anatomy of the cranial complex were viewed and debated for years thereafter [3, 19, 23–33]. During the nineteenth century, questions of form and function became rooted in comparative embryology, especially around the anatomical discoveries of workers like Rathke, Reichert, and Huxley, and the proposed laws of Haeckel [16, 18, 34, 35]. For example, Haeckel used his observations on the pharyngeal arches of various embryos to help explain how ontogeny could connect the forms of animals in a phylogenetic progression. Although Haeckel and his followers concluded rather erroneously that “ontogeny recapitulates phylogeny” [36], such early work built a vocabulary and an intellectual framework through which the mechanisms of structural and functional integration in the head could be probed for almost 200 years and up to the present.

**Fig. 1:**
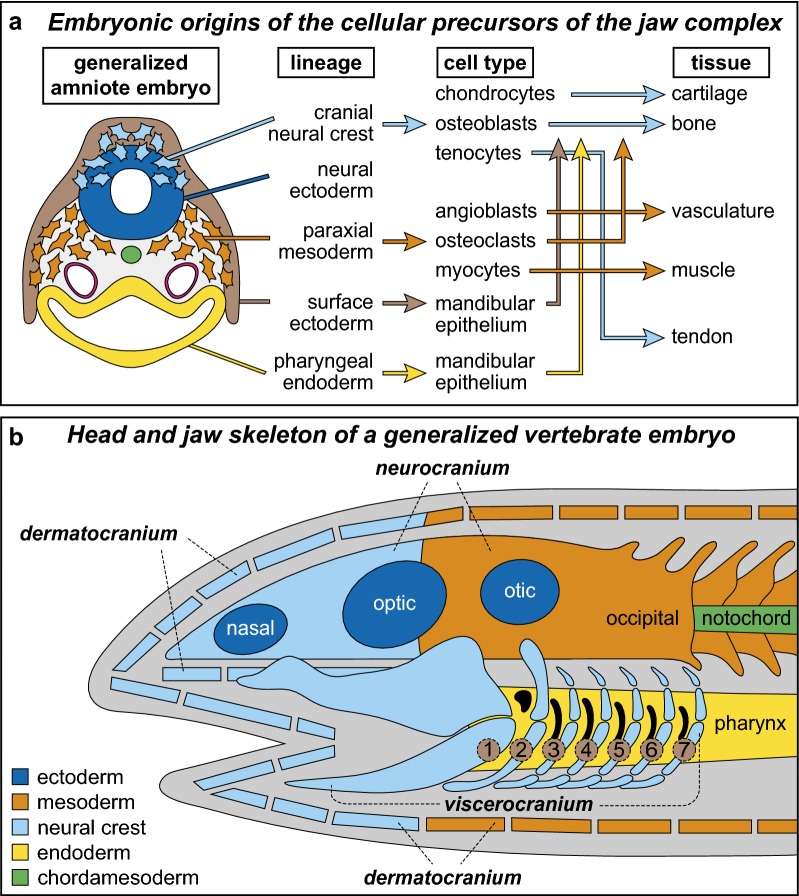
The embryonic origins of the jaw are highly conserved across amniotes despite species-specific differences in form and function. **a** Schematic transverse section through the midbrain-hindbrain boundary of a generalized amniote after neurulation showing the major lineages of cells and their cell types, cell–cell interactions (vertical arrows), and tissue derivatives that contribute to the jaw apparatus. **b** Head and jaw skeleton of a generalized vertebrate embryo showing the spatial arrangements of the neurocranium, viscerocranium, and dermatocranium. The neurocranium forms first as cartilage and surrounds the brain and sense organs such as in the nasal, optic, and otic capsules. The viscerocranium is the cartilaginous skeleton of the jaws and of the serially repeated arches (numbers 1 to 7) along the pharynx. The first arch is the mandibular arch, which consists of the palatoquadrate cartilage above and Meckel’s cartilage below. The second arch is the hyoid arch. The dermatocranium consists of the palatal, cranial vault, and tooth-bearing elements around the oral cavity. The viscerocranium is derived almost exclusively from NCM whereas the neurocranium and dermatocranium arise from both NCM and mesoderm (Modified and adapted from [22, 33, 38, 44, 75, 326, 395])

Yet while the evolutionary history and comparative anatomy of the jaws have been well characterized, many questions remain as to how individual components arise during development and achieve their requisite form and function. Derivatives of all three germ layers (i.e., ectoderm, mesoderm, endoderm), but especially the cranial neural crest mesenchyme (NCM), which is a major contributor to the jaws, must communicate seamlessly to produce a musculoskeletal system that is structurally integrated in support of its normal and often highly specialized use. Achieving such species-specific form and function in the jaws is a dynamic multidimensional problem that embryos have to solve [37]. In particular, there need to be mechanisms in place facilitating the species-specific modulation of parameters such as cell cycle length, cell size, cell number, cell specification, cell fate, cell differentiation, and more [7, 38–43]. Teasing apart such mechanisms as well as those underlying the migration, distribution, and interactions among jaw precursor populations (Fig. 1a), and also identifying the critical signals through which these cells acquire and implement their axial orientation, anatomical identity, and tissue type, is essential for understanding how the jaws become patterned and structurally integrated. By applying modern experimental strategies, the molecular and cellular events that underlie jaw form and function during development, disease, and evolution are being elucidated. Some of these studies and their key insights are reviewed in the sections below.

## Anatomical organization and integration of the jaw apparatus

The head skeleton has classically been organized into three compartments each with distinct embryological and evolutionary histories, anatomical locations, and various degrees of structural and functional integration: the neurocranium, viscerocranium, and dermatocranium (Fig. 1b) [3, 15, 19, 44–47]. The neurocranium has been defined as the skeleton that primarily forms first as cartilage and surrounds the brain and sense organs. The viscerocranium (or “splanchnocranium”) has been viewed as the cartilaginous skeleton of the jaws and of the serially repeated arches in the pharyngeal region of the gut tube. The neurocranium and viscerocranium are thought to have evolved as part of a vertebrate endoskeleton [3, 22, 48–50]. In contrast, the dermatocranium has been described as a component of the vertebrate exoskeleton, which in the skull consists of the palatal, cranial vault, and tooth-bearing elements around the oral cavity [46, 51–54]. Moreover, these skeletal systems have divergent embryonic origins in terms of cell lineages and process of differentiation [19, 37, 47, 50, 55, 56].

In jawed vertebrates, the neurocranium and dermatocranium develop from dual mesenchymal lineages (i.e., mesodermal mesenchyme and NCM), whereas the viscerocranium forms predominantly from NCM [54, 57–70]. Some aspects of the more posterior viscerocranial cartilages, such as in the laryngeal skeleton also appear to have contributions from mesoderm in amniotes [63, 71–73] and anamniotes [74, 75]. For the most part, the primary cartilages of the neurocranium and viscerocranium typically get replaced by bone through endochondral and perichondral ossification. Such bones are termed “cartilage bones” [3, 35, 51]. In contrast, most skeletal elements associated with the dermatocranium are not pre-formed in cartilage but arise principally as condensations of NCM and/or mesodermal mesenchyme that differentiate directly into “dermal bone” through intramembranous ossification [15, 19, 46, 51, 54, 62, 76–80]. However, these definitions are not exclusive as there are some endoskeletal bones that ossify intramembranously (e.g., “membrane bones”) and some exoskeletal bones that develop in conjunction with cartilage (e.g., “secondary” or “adventitious cartilage” of birds and mammals) [50, 51, 80].

During intramembranous ossification, mesenchymal cells condense and secrete a dense extracellular matrix, called osteoid, which is rich in collagen I and other fibers [81, 82]. Shortly afterward, osteoid mineralizes by incorporating calcium phosphate crystals that are absorbed from the vasculature and which provide rigidity to the fibrous network. During cartilage formation, mesenchymal cells condense and secrete an extracellular matrix rich in collagen II and other fibers to produce an avascular tissue [51, 80, 83, 84]. This process causes a tissue expansion such that chondrocytes become separated by vast amounts of extracellular matrix. Typically, as chondrocytes mature, they undergo apoptosis, vasculature invades the cartilage and brings in mineral, and the cartilage template is replaced by bone through endochondral ossification [79, 80, 85]. Despite these differences in how they differentiate, elements that transform from cartilage to bone via endochondral and perichondral ossification, and bones that arise directly through intramembranous ossification, become seamlessly integrated both structurally and functionally among the neurocranium, viscerocranium, and dermatocranium.

The amniote jaw skeleton contains elements from the viscerocranium and dermatocranium. The viscerocranial elements are derived from the pharyngeal arches, which are transient embryonic structures that produce upper and lower skeletal portions, as well as associated muscular, nervous, and circulatory elements [15, 19, 86, 87]. The jaws proper arise within the first pharyngeal arch, which is the mandibular arch. There has been considerable debate as to the boundaries between the mandibular arch and the region more anterior (i.e., “premandibular”), and also the extent to which the mandibular arch is in fact serially homologous with the other pharyngeal arches based on differences in the embryology and early patterning events of the oral cavity versus the pharynx [88–94]. Thus, some have suggested using terms like “oropharyngeal” to reflect these differences [19, 95].

**Fig. 2:**
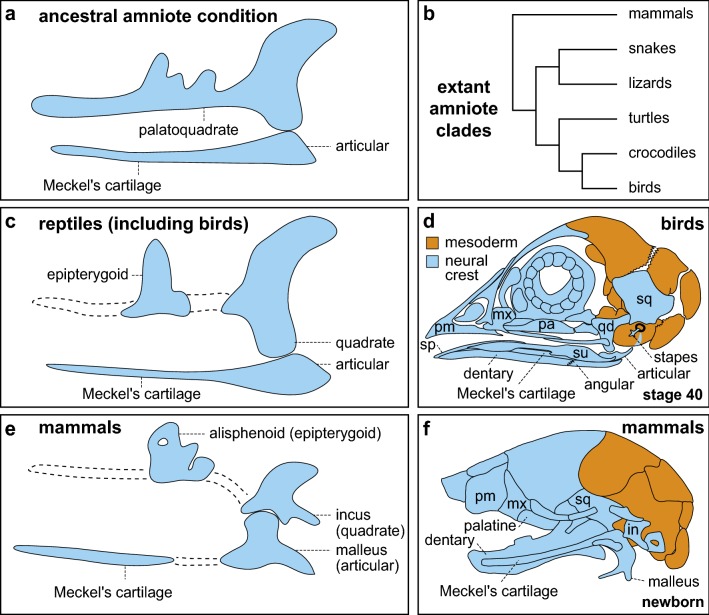
The amniote jaw skeleton has undergone evolutionary diversification in form and function. **a** Development of upper and lower cartilaginous elements of the mandibular arch in a generalized ancestral amniote. The cartilaginous upper jaw of an ancestral amniote consists of the palatoquadrate cartilage, which is a single element. The lower jaw contains Meckel’s cartilage with the articular cartilage at the most proximal end. **b** During the radiation of amniotes into the major clades of reptiles (including snakes, lizards, turtles, crocodiles, and birds) and mammals, the ancestral amniote condition became modified. **c** In reptiles and birds, all that persists of the palatoquadrate is the epipterygoid and quadrate cartilages that develop along condensations of NCM (dashed lines). The quadrate is the upper portion of the jaw joint and the articular cartilage is the lower portion. **d** In place of the palatoquadrate, the functional upper jaw of reptiles and birds is composed of dermal bones including the premaxilla (pm), maxilla (mx), and palatine (pa). The lower jaw is also made up of several dermal bones that surround Meckel’s cartilage including the dentary, surangular (su), angular, and splenial (sp). The amniote jaw skeleton is derived entirely from neural crest mesenchyme (NCM; light blue) whereas some elements in the skull roof are derived from mesoderm (orange). **e** In mammals, the epipterygoid contributes to part of the alisphenoid bone and the quadrate becomes the incus (in), which is an ossicle in the middle ear. In mammals, the articular cartilage becomes the malleus (ma) in the middle ear. **f** Mammals evolve an entirely new jaw joint between the dentary and squamosal (sq) bones, as the incus (in) and malleus (ma) become incorporated into the middle ear. The lower jaw is reduced to a single bone, the dentary (i.e., mandible) (Modified and adapted from [19, 37, 73, 102, 138, 140, 396])

In an influential but rather speculative hypothesis, the evolutionary origin and diversification of the vertebrate jaws were claimed to be tied to the emergence and elaboration of NCM, and a shift from passive, sessile feeding to active modes of predation [96–98]. While clearly the NCM (along with epidermal thickenings called placodes) have been essential to the success of vertebrates, vertebrates were likely active feeders long before they evolved jaws [99]. Nonetheless, after the jaws emerged in basal vertebrates, many of the same anatomical units and constituent parts have remained conserved across the various lineages including amniotes, albeit with some modifications and exceptions [3, 15]. In a generalized common ancestor for amniotes, the upper skeletal portion of the jaw (i.e., viscerocranial) contained the palatoquadrate cartilage while the lower portion consisted of Meckel’s cartilage (Fig. 2a). During the evolution of modern amniotes (Fig. 2b), however, these two cartilages no longer become the main contributors to the functional adult jaws. In reptiles and birds, the palatoquadrate is divided into two distinct cartilages, the epipterygoid and the quadrate (Fig. 2c) [100]. Generally, the epipterygoid contributes to the side of the braincase while the quadrate suspends the jaw skeleton from the temporal region of the skull [101–103]. In place of the palatoquadrate, the functional upper jaw of amniotes is made up of dermal bones from the dermatocranium, including the premaxilla, maxilla, quadratojugal, palatine, and pterygoid (Fig. 2d) [3, 45, 104].

In the lower jaw, Meckel’s cartilage typically persists as a cylindrical rod that rarely goes on to ossify [3, 105–107]. The lower jaw of reptiles and birds is also made up of several separate dermal bones from the dermatocranium that surround Meckel’s cartilage including the dentary, surangular, angular, and splenial (Fig. 2d) [100, 108–110]. Distinct from these dermal bones, the articular cartilage ossifies within the proximal portion of Meckel’s cartilage and contacts the quadrate cartilage to form the jaw joint. Thus, the actual connection between the upper and lower jaws of reptiles and birds comes from two ossified remnants (i.e., quadrate and articular) of the ancestral viscerocranial upper and lower portions of the first oropharyngeal arch [102]. This also typifies the jaw joint for all non-mammalian jawed vertebrates.

In contrast, the mammalian jaw skeleton is highly derived from the ancestral amniote condition. First, the homolog of the epipterygoid helped close off the expanded mammalian braincase by giving rise to a portion of the mammalian alisphenoid bone (Fig. 2e) [111–114]. Second, the functional lower jaw went from having up to six different bones to a single bone, the dentary (i.e., mandible) (Fig. 2f) [45, 115]. Third, the quadrate became reduced in size, no longer participated in the jaw joint, and evolved into the incus, which is one of the mammalian middle ear ossicles [116–118]. During this evolutionary transformation, the articular, like the quadrate, became modified into another middle ear ossicle, the malleus [117]. In association with the viscerocranial jaw joint becoming middle ear ossicles, a new jaw joint formed in the dermatocranium between the dentary and squamosal bones (i.e., the dentary-squamosal or temporal-mandibular joint) of mammals [119, 120]. Such a transformation demonstrates how jaw bones can be repurposed to have new functions [121–123]. In this case, bones that once supported feeding become bones for hearing [117, 118, 120, 124]. So, while in reptiles and birds, the bones that conduct sound to the inner ear remain closely associated with the jaw, in mammals, the sound-conducting middle ear ossicles become isolated from the jaw joint and encapsulated within the skull [125]. Such an arrangement apparently confers mammals with an efficient auditory system capable of detecting high frequencies and protected from the masticatory apparatus [126].

In general, jaw movement is enabled by pairs of adductor, abductor, and levator muscles that insert onto various aspects of the mandible [127–129]. The main jaw adductor muscles are innervated by the trigeminal nerve (n. V) whereas the abductors are innervated by the facial nerve (n. VII) [130]. For most amniotes, lateral movement is fairly restricted and jaw adduction and abduction occurs at the parasagittal plane. The mammalian jaw adductor complex underwent significant rearrangement and modification in association with the evolution of mastication and presumably the need to increase bite force [123, 131]. Mammals also evolved a novel series of pharyngeal elevators and constrictors to support their unique swallowing and suckling behaviors [95, 132].

As in the rest of the musculoskeletal system, the muscles and bones of the jaw are joined by tendons, which are continuations of the connective tissue fascia that ensheath skeletal muscles. In contrast to muscle and bone, which are well vascularized, tendons are avascular. Tendons primarily distribute tensile forces from muscle to bone and the junction between tendon and bone, called an enthesis, is marked by a transition zone between the fibrous matrix of tendon and the mineralized matrix of bone [133–135]. Bundles of densely packed and axially aligned fibers (i.e., Sharpey’s) that comprise tendons must smoothly transform into cortical bone for effective transmission of mechanical loads. Moreover, fibrocartilage may develop within compressed regions when tendon is wrapped against the surface of bone, which can help create a gradient in material properties along the transition from soft to hard tissues (i.e., tendon to bone) and dissipate the stress concentration at the bony interface [136]. The hallmarks of fibrocartilaginous tendons include sparsely distributed chondrocytes and a cartilaginous matrix enriched with molecules associated with resisting compression [137].

In order for each of the cartilages, bones, muscles, and tendons of the amniote jaw complex to attain proper form, achieve structural integration, and become functionally enabled, their precursor populations must acquire and/or act upon multiple dimensions of patterning. These dimensions include developing with the appropriate cell and tissue type (e.g., chondrocyte, osteoblast, myocyte, tenocyte), axial orientation (e.g., dorsal–ventral, rostral–caudal, proximal–distal, medial–lateral), anatomical identity (e.g., upper jaw versus lower jaw), and species-specific size and shape (e.g., mouse-like versus human-like, or quail-like versus duck-like) [37, 42, 138]. Clearly, each component within the jaw complex can be transformed rapidly and dramatically during the course of amniote evolution (and also in the case of birth defects), and undoubtedly this occurs via changes in the molecular and cellular programs that underlie the multiple dimensions of patterning. However, such changes must be constrained on one level or another because over the long run, the essential internal relationships among the various musculoskeletal elements have to be maintained with high fidelity and in a manner that meets any necessary functional demands. How this happens could be considered an emergent property of all the signaling pathways and gene regulatory networks that are deployed over time across three-dimensional space, as well as the embryonic histories and iterative interactions of every contributing cell and tissue. In this regard, the developmental biology of the jaw apparatus seems almost infinitely complicated to sort out. Nonetheless, good progress can be made in characterizing the multidimensional and dynamic system that generates the amniote jaw complex by focusing on the hierarchical levels of anatomic and embryonic organization, by identifying common principles, and by emphasizing fundamental molecular and cellular mechanisms.

## Cellular origins of musculoskeletal tissues in the jaw

**Fig. 3:**
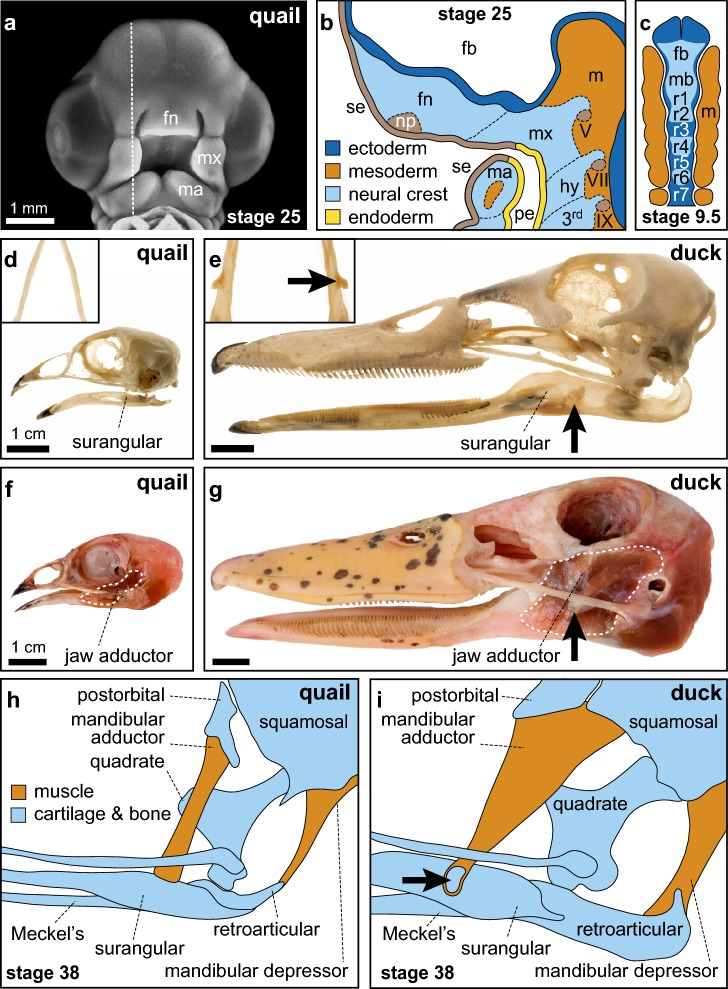
The development of the amniote jaw complex involves critical contributions from multiple embryonic populations. **a** Frontal view of stage 25 quail embryo. The frontonasal (fn), maxillary (mx), and mandibular (ma) primordial are visible (dotted line indicates the sagittal section plane for **b**). **b** By stage 25, the frontonasal (fn), maxillary (mx), mandibular (ma), and hyoid (hy) primordia (sagittal view) are populated by NCM (light blue) surrounded by surface ectoderm (se; tan), pharyngeal endoderm (pe; yellow), and forebrain neuroepithelium (fb; dark blue) and contain contributions from neural crest, nasal placode (np), and cranial ganglia (V, VII, IX). Mesoderm (m) that produces skeletal tissues is distributed caudally. **c** Prior to migration, at stage 9.5 (dorsal view) cranial NCM (light blue) delaminates from the forebrain (fb), midbrain (mb), and hindbrain rhombomeres (r; dark blue). Cranial NCM migrates alongside paraxial mesoderm (m; orange). **d**, **e** Head skeleton of adult quail and duck. The duck surangular bone, which lies dorsal to the dentary bone along the lower jaw (inset), contains a robust coronoid process (black arrow) along its lateral margin that is absent in quail. **f**, **g** The mandibular adductor muscles (white dashed outline), which close the jaw, are relatively larger in ducks than in quails. The caudal external mandibular adductor muscle originates posterior to the orbit and inserts laterally on the duck coronoid process (black arrow). This muscle is relatively smaller in quails and inserts along the dorsal margin of the surangular. **h** By stage 38 in quails, the narrow mandibular adductor muscle (orange) inserts dorsally onto the coronoid process of the surangular bone (light blue). **i** By stage 38 in ducks, the broad mandibular adductor inserts laterally onto the coronoid process and contains a secondary cartilage (arrow) within the tendon enthesis (Modified and adapted from [6, 19, 37, 138, 140, 233, 283])

The cartilages and bones in the upper and lower portions of the amniote jaws arise within embryonic prominences flanking the stomodeum, or presumptive oral cavity (Fig. 3a). The first oropharyngeal arch (i.e., mandibular arch) contains two pairs of prominences: the maxillary processes, which lie lateral to the stomodeum and give rise to the secondary palate and portions of the upper jaws; and the mandibular processes, which lie inferior to the stomodeum and produce the lower jaws [139, 140]. Additional prominences, specifically the frontonasal process in reptiles and birds, and the lateral and medial nasal processes in mammals, give rise to the mid and upper face and the primary palate [141–144]. Modulating growth and other parameters in these prominences enables diverse and complex morphologies to develop and evolve, but abnormal variation often causes facial and palatal clefting, which are some of the most common human birth defects [144–152].

The oropharyngeal arches are populated by NCM (Fig. 3b, c), which arises at the boundary between the neural plate and the non-neural ectoderm following an epithelial to mesenchymal transition [153–159]. NCM migrates extensively and produces numerous cell types in the jaw apparatus including all the chondrocytes that make cartilage, osteoblasts that make bone, tenocytes that make tendon, and ligamentous fibroblasts that make other muscle connective tissues (Fig. 1a) [19, 54, 62, 66–69, 77, 160–164]. NCM appears to be drawn from the neural tube to the oropharyngeal arches via chemoattractant gradients. Many molecules like fibroblast growth factors (FGF), vascular endothelial growth factors (VEGF), and other cytokines and secreted proteins are thought to attract migrating NCM, but whether such gradients are sufficient to guide long-range NCM migration remains an open question [165–169]. Other in vivo and in silico data predict that a chemoattractive gradient may not be required for collective NCM migration. Instead, contact inhibition may drive the long-range, directional migration of NCM [153, 169–172]. Repulsive signals also steer streams of migrating NCM by way of Eph/ephrin and neuropilin/semaphorin signaling for example [173–175]. Likely a combination of contact inhibition along with attractive and repulsive signals regulates cranial NCM streaming and funnel NCM into their proper oropharyngeal destinations where they eventually differentiate as an array of interconnected jaw tissues.

Although NCM differentiates into many cell and tissue types, the extent of their initial developmental potency has been disputed. Conflicting interpretations of clonal analyses and lineage tracing experiments have obscured whether NCM is truly multipotent, or whether NCM is a diverse population of fate-restricted cells [162, 176–182]. For instance, studies using fluorescent “confetti” reporter mice reveal that individual migratory neural crest cells commonly contribute to many cell types and multiple tissues and suggest that NCM is indeed multipotent [183]. Correspondingly, the gene regulatory networks that direct NCM toward differentiation have become much better understood [184–188] and undoubtedly their continued delineation will help clarify the multifaceted genetic underpinnings of neurocristopathies, which often have widespread and debilitating effects [189–191].

Besides NCM, the development of the jaw complex also involves critical contributions from non-neural ectoderm and pharyngeal endoderm, which form the epithelia that surround the mandibular arch, as well as from paraxial mesoderm (Fig. 3b, c). Epithelial tissues derived from the non-neural ectoderm include placodes that produce tissues like olfactory epithelium in the nasal capsule as well as cranial ganglia like the trigeminal that support the innervation of the mandibular arch [194–199]. The epidermis, which becomes stratified into multiple layers, likewise comes from the non-neural ectoderm and produces the enamel of teeth [46, 76] as well as the keratinized portions of jaw structures such as horns, beaks, and egg teeth [193, 200–204]. Paraxial mesoderm gives rise to angioblasts that build blood vessels, osteoclasts that resorb bone, and myocytes that make skeletal muscle in the jaws [63, 71, 128, 161, 205–211].

Cranial skeletal muscles are distinct from trunk muscles in terms of the organization of their embryonic precursor populations and the gene regulatory networks that govern their differentiation [128, 205, 213–221]. Amniote jaw muscles derive from unsegmented populations of paraxial mesoderm [128, 161, 71, 206, 209] whereas in the trunk, skeletal muscles arise from paraxial mesoderm that is organized into segmented somites [222–225]. These differences not only reflect the complex developmental and evolutionary histories of the head but also seem to influence the patterns of muscle gene expression. While transcription factors like *Mrf4*, *Myf5*, *MyoD*, and *Myogenin* are required for myogenesis throughout the body, the specific subsets of genes and the genetic hierarchy regulating these factors vary between cranial muscle groups [205]. For example, jaw muscles employ a suite of genes that is distinct from trunk muscles and even other cranial muscles [217, 218, 226]. Some signals like those from the bone morphogenetic protein (BMP) pathway repress muscle differentiation in both the head and trunk, while Sonic Hedgehog (SHH) and Wingless (WNT) signaling promotes muscle differentiation in the trunk but inhibits muscle differentiation in the head [216]. Specifically, connective tissues surrounding head muscles express antagonists like *Gremlin* and *Frizzled-related protein (Frzb)*, which relieve repression of muscle development by BMPs and WNTs, respectively, and allow cranial muscles to differentiate.

The above example involving BMP and WNT signaling illustrates one of the many ways the patterning and differentiation of cranial skeletal muscle rely upon signals emanating from adjacent NCM-derived connective tissues. Myogenic precursors migrate alongside NCM *en route* to the first and second oropharyngeal arches [55, 161, 71, 227, 228] and multiple aspects of jaw muscle pattern are regulated by NCM-derived connective tissues such as fiber type, muscle orientation, and the precise locations of attachments [128, 205, 212, 218, 226, 229, 230]. This intimate spatial and temporal relationship is similar to what occurs in the trunk [225] where connective tissue fibroblasts (although these instead arise from trunk mesoderm) supply critical signals for both fast- and slow-twitch muscle differentiation and lay down the basic muscle patterns prior to tendon differentiation [231, 232].

Not only do such developmental interactions between NCM and mesodermal mesenchyme ensure the structural integration necessary for achieving appropriate muscle function during ontogeny, but they also seemingly help maintain the co-evolution of the musculoskeletal system throughout phylogeny. This conclusion is buttressed by results from chimeric transplant experiments that exploit the different jaw morphologies of quails and ducks (Fig. 3d–g). In particular, quail–duck chimeras have revealed the ability of NCM-derived tendon and muscle connective tissues to dictate the species-specific attachments of jaw muscles that have evolved in connection with the distinct modes of feeding that characterize each of these birds [6, 233]. For example, transplanting pre-migratory NCM from quail to duck embryos produces duck-host-derived muscles with quail-like shape and attachment sites [6]. Such mechanistic reliance of the jaw muscles on their associated connective tissues during development likely underlies the capacity of species to adapt by co-evolving their musculoskeletal system in ways that often seem astonishingly well suited for novel functions.

## Epithelial interactions underlying jaw patterning and differentiation

Despite the wide variety of highly specialized jaw morphologies, the basic Bauplan and the underlying genetic modules of the developing jaw complex remain relatively conserved across amniotes. All amniote jaws are oriented such that the most proximal components articulate at a hinge even though the distal components may vary greatly in length and in form. To establish the correct positional information along the axes of the developing jaw skeleton, the mandibular arch relies upon discrete and nested molecular programs that are regulated by and affect the NCM. One elegant hypothesis to explain this phenomenon is known as the “hinge and caps model” wherein two appositional units (i.e., upper jaw and lower jaw) are thought to maintain their own intrinsic polarity through a patterning system that reflects the competence of NCM to respond to an array of positionally located epithelial signals [234]. In this context, species-specific changes to protein coding sequences, ligand and receptor expression domains, duration of gene expression, and/or sensitivity to signaling could allow the proportions and relative positions of skeletal elements to change along the proximodistal axis during evolution while simultaneously maintaining the basic “hinge and caps” organization of the jaws [38, 39, 235–239].

Numerous studies have shown that the signals from the epithelium are spatially and temporally dynamic and, in response, NCM expresses a combinatorial suite of transcription factors such as the *Msx*, *Dlx*, *Prx*, *Hand*, *Six*, *Bapx*, and *Barx* families, which in turn affects the anatomical identity of the maxillary and mandibular prominences [173, 240–260]. For example, perturbing *Dlx* gene expression transforms maxillary into mandibular jaw bones [243, 244, 249]. Such homeotic transformations caused by disruptions to homeobox genes like *Dlx* and others demonstrate that in general the stereotypic and programmatic responses of transcription factors, which are elicited by signals from adjacent epithelia, are a keystone of jaw morphogenesis. This is not unlike what happens along the anteroposterior axis of the trunk or the proximodistal axes of the limbs, which are patterned by overlapping expression domains of *Hox*-family transcription factors. However, a seemingly important difference is that the frontonasal process as well as the maxillary and mandibular primordia of the first oropharyngeal arch (unlike the more posterior arches such as the hyoid arch) are *Hox* free and, thus, they are reliant on different gene regulatory networks and signaling interactions to guide their morphogenesis [261–264].

One of the primary functions of these epithelial–mesenchymal signaling interactions is to establish axial polarity in the face and jaws. For example, to set up the dorsoventral axis of the upper jaw, retinoic acid (RA) signaling triggers a sequence of reciprocal signaling events among the neuroepithelium, NCM, and surface ectoderm [265–267]. Epithelial–mesenchymal signaling between the NCM and the surface ectoderm defines a signaling center called the frontonasal ectodermal zone (FEZ) that consists of complementary *Fgf8* and *Shh* domains separated by a precise boundary [143, 268]. RA signaling maintains *Fgf8* and *Shh* expression domains in both the neuroepithelium and surface ectoderm [265, 269]. Rotating the FEZ 180° induces ectopic *Fgf8* and *Shh* domains, extra dorsoventral axes, and supernumerary structures of the upper jaws such as duplicated cartilages and egg teeth in birds [141, 268].

Likewise, the anteroposterior axis of the jaw skeleton is established through interactions between NCM and the pharyngeal endoderm, which also relies on *Shh* expression to establish polarity and support cartilage development [79, 270–273]. Ablating localized regions or altering the growth of pharyngeal endoderm prevents formation of the quadrate, Meckel’s cartilage, the articular, and the hyoid [274–276]. Rotating pharyngeal endoderm by 90°, 180°, or 270° leads to ectopic and correspondingly re-oriented cartilaginous elements. Finally, in terms of the mediolateral axis, ectopic midline structures like egg teeth can be induced in the lateral nasal process by simultaneous local inhibition of BMP signaling and the administration of exogenous RA, which presumably mimics the local signaling environment of the frontonasal process [245, 266]. These experiments and many others underscore the critical role of epithelia and their cadre of secreted factors in establishing the axes of the jaw skeleton and ultimately the relative positions of individual jaw bones and cartilages [147, 148, 237, 265, 277–279].

As part of its genetic response to the epithelial interactions that establish the major axes and anatomical identity of skeletal elements along the jaws, NCM executes intrinsic developmental programs that impart individual cartilages and bones with species-specific size and shape. Such insight comes mostly from interspecific transplant experiments involving the embryos of salamanders, frogs, birds, and mice, which have shown that this aspect of patterning in the jaws is largely driven autonomously by the NCM [37, 39, 40, 42, 156, 280–286]. Chimeric model systems have also enabled mechanisms underlying the complex interactions between NCM and surrounding epithelial tissues to be interrogated on the molecular level. For instance, transplanting quail NCM into a duck host produces a smaller jaw with quail-like, species-specific morphology [7, 283, 287]. Such a complex morphological transformation is driven by NCM-mediated temporal and spatial changes in the expression of genes known to be involved in the patterning, differentiation, and growth of the jaw skeleton such as members and targets of the BMP, FGF, SHH, and transforming growth factor beta (TGFβ) pathways [38, 40, 283, 287, 288]. Furthermore, NCM seems to be remarkably pliant and, for example, can even follow cues from the local developmental environment that normally pattern mesoderm-derived skeletal elements [114]. These experimental findings serve as a testament to the regulatory abilities, developmental plasticity, and evolutionary significance of the NCM during jaw evolution [7, 19, 37, 39, 96, 138, 140, 164, 191, 289].

Moreover, the use of an anatomically diverse range of model systems (especially avian) has enabled the developmental programs responsible for evolutionary changes to the dimensions of the jaw skeleton to be elucidated [290]. For example, studies involving Darwin’s finches and other birds including chicks, ducks, quails, and cockatiels have not only uncovered components of genetic modules and/or gene regulatory networks that specify the axes of the jaw skeleton but have also helped elucidate how changes to these components can generate species-specific variation in depth, width, and length during evolution. In particular, BMP signaling affects depth and width whereas calcium signaling affects length [291–295]. Species-specific jaw length also appears to be dependent on NCM-mediated expression of enzymes involved in bone resorption such as *matrix metalloproteinase 13 (Mmp13).* In this case, quail embryos express high levels of MMP13 in the NCM-derived jaw skeleton while duck embryos express relatively little, and inhibiting MMP13 in quail embryos lengthens the jaw [38]. TGFβ and WNT signaling also appears to regulate the size and shape of the upper jaw [236]. Similarly, sequence changes in transcription factors like *Alx1* also affect species-specific jaw shape [296]. Finally, thousands of putative active enhancers seem to be operating during craniofacial morphogenesis indicating that there are many yet to be discovered mechanisms from paracrine signaling to transcriptional regulation likely governing the evolutionary diversification of jaw size and shape [297, 298].

Not only does the initial patterning of NCM in terms of axial orientation and anatomical identity require numerous reciprocal signaling interactions with adjacent epithelia but also the differentiation of NCM into skeletal tissues such as bone depends on these interactions as well [138, 288, 299]. For example, intramembranous ossification of the lower jaw requires precisely timed, reciprocal interactions with overlying epithelium. Surgically removing mandibular epithelium prevents NCM from forming bone [288, 299]. However, there does not seem to be anything intrinsically osteogenic about mandibular epithelium since NCM can still make bone in the mandibular primordia even when interacting with epithelium from the forelimb [300]. These and other tissue recombination experiments reveal that NCM helps establish the location of osteogenesis during jaw development likely through some yet to be identified instructive signals.

NCM also controls the timing of mandibular osteogenesis. If mandibular epithelium is removed at an early stage, then jaw bone fails to form. However, at a slightly later stage (presumably after some critical signaling events between NCM and the mandibular epithelium have occurred) bone can form in the absence of the epithelium [288, 299]. While on the surface this would suggest that the epithelium determines when bone forms, quail–duck chimeras demonstrate that the precise timing of this epithelial–mesenchymal interaction and ultimately the induction of bone is reliant upon an NCM-mediated developmental program involving BMP signaling [288]. Quail embryos develop faster than duck embryos due to intrinsic differences in their rates of maturation (17 versus 28 days from fertilization to hatching). When NCM is transplanted unilaterally from quails to ducks, the entire program for osteogenesis is accelerated and precocial bone forms on the quail-donor side three developmental stages earlier than on the contralateral duck-host side [40]. Additionally, in chimeras, bone can form much sooner in the absence of epithelium coincident with the presence of faster-developing quail donor NCM [288]. NCM appears to accomplish this task by using BMP signaling to govern the timing of interactions with epithelium as well as jaw bone formation. The ability of NCM to exert control over the location and timing of key osteogenic events as well as the regulation of critical signaling pathways provides another crucial insight into how NCM acts as a fundamental developmental mechanism linking the species-specific evolution of form with function in the amniote jaw skeleton.

## The role of mechanical forces in jaw form and function

During embryogenesis, the formation and growth of jaw tissues are also influenced by external factors, including the mechanical environment. Throughout the body, muscles, bones, and tendons respond and adapt to mechanical stimulation via various mechanotransduction pathways, often undergoing hypertrophy in the presence of increased loading, and atrophy with disuse [136, 137, 301–305]. In sites where tendons transduce high magnitude forces from muscles, bony eminences may form. Pools of cells which express both cartilage (e.g., *Sox9*) and tendon (e.g., *Scx*) lineage markers contribute to bony eminence development in the head and trunk such as the angular process of the mandible, deltoid protuberance of the humerus, and great trochanter of the femur [306, 307]. In this way, achieving proper musculoskeletal pattern, structural integration, and linkage between form and function depends on the dynamic ability of tendons and other tissues to detect and respond to biomechanical cues in the local environment. Such developmental plasticity in response to mechanical forces helps shape the jaw skeleton and creates robust muscle attachments. For these reasons, gaining a deeper understanding of the molecular and cellular mechanisms that allow certain tendons to achieve robust osseointegration might some day help enhance the capacity of torn muscle insertions to be re-attached to bone or even regenerated in clinical situations via molecular therapies [308–311].

The primary source of biomechanical forces that contribute to jaw development is embryonic motility. As neuromuscular junctions form, they facilitate spontaneous muscle contractions and cause embryos to move various parts of the skeleton. Presumably, embryonic motility feeds directly into a cascade of molecular and cellular events [137, 233, 312–317] that ultimately enable embryonic form to presage adult function. Birds have served as a well-suited model system for characterizing and quantifying embryonic motility because their relatively large embryos are easily accessed and observed [42, 318–326]. In chicks, the first neuromuscular junctions form in the trunk [327]. Random depolarizations strengthen neuromuscular junctions and mature into cyclic, stereotyped movements of the head, jaws, trunk, and limbs. As Wolff’s Law predicts, disruptions to embryonic motility cause widespread and severe musculoskeletal defects. Early paralysis can lead to abnormal joint cavitation [323–331]. Later paralysis can alter the size, shape, extent of ossification, and relative proportions of skeletal elements [303, 305, 332–334]. However, mechanisms that facilitate the relationship between mechanical stimulation and musculoskeletal pattern have for the most part remained obscure.

One mechanically responsive skeletal tissue that appears to be unique to amniotes and plays a critical role in the proper form and function of the jaw is secondary cartilage. Secondary cartilage develops independent of, and subsequent to, the primary cartilaginous skeleton (e.g., the neurocranium and viscerocranium) [3, 51, 335, 336]. Secondary cartilage is found within cranial joints, the sutures of some calvarial bones, the clavicles, antlers of deer, certain ligaments and tendons, and the transient calluses that arise during the healing of broken bones [42, 80, 85, 233, 337–340]. While secondary cartilage is now limited to birds and mammals, there is some fossil evidence suggesting that a non-avian dinosaur possessed secondary cartilage within the mandibular adductor insertion, raising the possibility that this tissue was also present in archosaurian reptiles more broadly [341, 342].

The formation of secondary cartilage relies on mechanical stimulation and, therefore, the evolutionary presence or absence of secondary cartilage reflects species-specific variation in functional jaw anatomy [336, 339, 343, 344]. In humans, rats, cats, and ducks, secondary cartilage forms at the tendon insertion (i.e., enthesis) of the jaw adductor muscles on the coronoid process (Fig. 3h, i) [45, 80, 233, 340, 345–350]. An equivalent secondary cartilage is absent in mice, guinea pigs, chicks, and quails [233, 346–354]. Why secondary cartilage arises at this location in some species and not others is unclear but presumably the underlying mechanisms are responsive to differential forces generated by muscle attachments and jaw movements [42, 137, 233, 312, 313, 315, 350]. In humans and ducks, a robust and protruding secondary cartilage at the coronoid process (that eventually becomes a bony process) provides a broad lateral insertion for the adductor muscles, which enhances leverage and facilitates the sliding motion needed for their specialized modes of feeding [355–362]. Ducks feed via a suction pump mechanism and the levered straining of water. This involves rapid opening and closing of the mandible, which requires sudden acceleration and significant force [356]. Conversely, in quails and chicks, which peck at their food and use the distal tips of their beaks like precise pincers, the adductor muscles insert dorsally and the coronoid process appears as a slight bony ridge (Fig. 3h) [109, 110, 201, 363–367].

As is the case for the jaws of other mammals, secondary cartilage at the human condylar and coronoid processes is required for proper kinetic movement of the temporal-mandibular joint (TMJ) [349, 357, 368, 369]. As described earlier, the TMJ is a uniquely mammalian articulation point for the upper and lower portions of the jaw that is not homologous to the quadrate-articular jaw joint of other vertebrates. The TMJ plays a critical role in normal mammalian jaw function and is especially reliant upon the secondary cartilage that covers its articulating surfaces. Secondary cartilage degeneration can often result from trauma, altered mechanical loading, genetic perturbations, and/or hormonal changes, and lead to temporomandibular disorders (TMD). TMD are pervasive human clinical conditions that affect approximately 10% of the population [370, 371] and cause acute pain and suffering for patients [372]. Strategies for molecular and cell-based therapies to restore normal TMJ function impaired by birth defects, injury, or disease can likely benefit by identifying mechanisms that control the development of secondary cartilage. However, mechanisms through which chondrogenic and mechano-responsive factors are regulated, and how changes to the mechanical environment alter expression of these factors remain unclear. Thus, elucidating how secondary cartilage is induced and maintained can provide an important example of how form and function become integrated during the development of the jaw skeleton and also can help shed light on a major unmet clinical need.

The exact nature of the mechanical forces and the downstream molecular mediators that induce and maintain secondary cartilage requires further elaboration. Secondary cartilage fails to form on the duck coronoid process following in ovo paralysis [42, 233], whereas ex vivo culture of embryonic chick jaws shows that cyclic mechanical stimulation is sufficient to promote secondary chondrogenesis at the joint between the quadrate and quadratojugal [312, 313]. Finite element models, which integrate embryonic motility with species-specific variation in jaw anatomy, have predicted that there are significant qualitative and quantitative differences in the local force environment leading to the presence of secondary cartilage on the duck coronoid process but not on that of the quail [233]. This is based primarily on the observation that in duck, the mandibular adductor inserts on the lateral aspect of the surangular bone, whereas in quail, the insertion is along the dorsal margin. Additionally, the duck insertion is also much more proximal to the jaw joint. Such geometries imply that duck embryos experience substantially higher and more heterogeneous shear stress concentrations at the mandibular adductor insertion, which at their maximum can be 60 times greater than those predicted for quail [42]. Also, based on cross-sectional area, the embryonic duck mandibular adductor has a maximum contractile force approximately 2.8 times  greater than that of quail. Importantly, chimeric “quck” (i.e., quail donor NCM transplanted into a duck host) form a quail-like jaw complex including a transformation of the lateral to dorsal insertion of the mandibular adductor muscle and a corresponding lack of secondary cartilage even though the mandibular adductor muscle itself comes from the duck host.

Thus, the lateral position of the insertion of the mandibular adductor muscle in ducks, which is established as a consequence of patterning by NCM-derived connective tissues [6, 233], seemingly creates a combination of axial tension and compression when the adductor muscle contracts and the jaw closes [42, 233]. By comparison, cells in the dorsal insertion of the mandibular adductors in quails likely experience primarily axial tension. These divergent mechanical environments presumably lead to the differential activation of mechano-responsive signaling pathways, which in turn produce cellular changes that in due course dictate the presence or absence of secondary cartilage on the coronoid process. Such results point to the indispensable contributions of NCM to establishing the species-specific form and function in the jaw apparatus. Moreover, some of the pathways required for derivatives of the NCM to adapt and respond to the mechanical environment are beginning to be better understood. Not surprisingly, the ways that developmental programs integrate biomechanical forces and the individual genes and cells that respond to cues from the mechanical environment appear to be context dependant and tissue specific. For example, WNT signaling and the osteocyte-specific WNT inhibitor, *sclerostin*, have been implicated in mechanosensitive bone remodeling [373–375]. Other mechanisms of mechanotransduction seem to include ligands being freed from the extracellular matrix, signaling through ion channels, changes in focal adhesions, and dynamic rearrangement of the cytoskeleton, among others [233, 302, 376–387].

The quail–duck chimeric system has been especially useful for further pinpointing molecular mechanisms through which jaw morphology and mechanical forces interact [42, 233]. For instance, members and targets of the FGF and TGFβ signaling pathways are differentially responsive to the species-specific variation in the mechanical force environment of quail versus duck. Both of these pathways are known to play a role during mechanotransduction and chondrogenesis in other biological contexts [388–394], and both pathways are required for secondary chondrogenesis at the coronoid process [42]. Moreover, exogenous FGF and TGFβ ligands can rescue secondary cartilage in paralyzed duck (again, when no secondary cartilage forms) and also induce cartilage in the quail mandibular adductor insertion, where normally there is none. These important mechanistic insights help explain how species-specific morphology, mechanical forces, and resultant changes in signaling activity become integrated and contribute to musculoskeletal plasticity in the jaw apparatus. In other words, the reason why secondary cartilage forms in some locations in some species and not others is likely because of the way embryonic motility interacts with NCM-mediated muscle pattern to create a qualitatively and quantitatively different mechanical force environment. Thus, this example illustrates that while form initially determines function, function can also serve as a forceful regulator of musculoskeletal form in the jaw complex during development and evolution.

## Conclusion

In 1916, E.S. Russell posed the question in his now classic book, *Form and Function* [18], “Is function the mechanical result of form, or is form merely the manifestation of function or activity? What is the essence of life, organisation or activity? (p.v).” A broad range of experimental strategies across different model systems have revealed that NCM is an essential player in most, if not all, of the decisive events that generate the primary organization of the amniote jaw complex. NCM not only provides the raw materials for the cartilages, bones, and other essential components that comprise the jaws, but NCM is also required for the critical signaling interactions that imbue these tissues with the multidimensional aspects of patterning from which their form is derived. Deficiencies in NCM or perturbing these interactions on the molecular or cellular level alters the form of the jaw complex in profound ways, which illuminates why the jaw complex is both highly evolvable and extremely susceptible to developmental defects [164]. Moreover, while NCM and neighboring epithelia typically collaborate to pattern the cartilages and bones of the jaws, and while NCM and mesodermal mesenchyme work together to pattern the jaw muscles, NCM seems to act as the dominant source of information that gives all of these jaw structures their species-specific size and shape. In this role, NCM is the common denominator that underlies the structural integration of the jaw apparatus, generates species-specific variation, and likely serves as a responsive target of natural selection during evolution [7, 37, 138, 140, 191]. Moreover, NCM has augmented the evolutionary potential (i.e., adaptability) of the pharyngeal and rostral portions of the head and imparts the jaw skeleton with developmental plasticity, as evidenced by the ability of the NCM-derived skeleton to respond to mechanical forces like in the case of secondary cartilage. Initially, the form of the jaw appears to dictate function, but then through embryonic motility, function modulates form. In other words, NCM sets up the species-specific “organisation” of the jaw apparatus prior to the onset of muscle “activity.” But once jaw activity starts, the form of the skeleton adapts to support its functional needs. The species-specific form of the duck jaw apparatus, especially the geometry of the NCM-mediated muscle attachments, produces mechanical forces that differentially regulate FGF and TGFβ signaling and cause secondary cartilage to form on the coronoid process. In this regard, NCM not only mediates form but also helps shape the biomechanical environment. Additionally, the patterning abilities and plasticity found in NCM-derived jaw progenitors facilitate seamless integration of form and function during embryonic development and evolution. These same processes are likely perturbed in cases of injury or disease. Overall, elucidating the molecular and cellular mechanisms through which NCM governs the species-specific patterning of cartilage, bone, tendon, and muscle has shed light on the evolutionary integration of form and function in the amniote jaw complex, and in the near future could help remedy an unmet clinical need to repair and regenerate jaw tissues affected by birth defects, disease, or injury.

## Data Availability

Not applicable.

## Abbreviations

BMP: bone morphogenetic proteins
FGF: fibroblast growth factors
FEZ: frontonasal ectodermal zone
FNP: frontonasal process
NCM: neural crest mesenchyme
RA: retinoic acid
SHH: sonic hedgehog
TMD: temporomandibular disorders
TMJ: temporomandibular joint
TGFβ: transforming growth factor beta
VEGF: vascular endothelial growth factors
WNT: wingless
